# USP13 promotes development and metastasis of high-grade serous ovarian carcinoma in a novel mouse model

**DOI:** 10.1038/s41388-022-02224-x

**Published:** 2022-02-16

**Authors:** Juntae Kwon, Hyeongjwa Choi, Anna D. Ware, Bernadette Cecilia Morillo, Haiyang Wang, Kerrie B. Bouker, Xiongbin Lu, Todd Waldman, Cecil Han

**Affiliations:** 1 Department of Oncology, Georgetown University School of Medicine, Washington, DC 20007 USA; 2 Biochemistry and Molecular Biology, Georgetown University School of Medicine, Washington, DC 20007 USA; 3 Lombardi Comprehensive Cancer Center, Georgetown University School of Medicine, Washington, DC 20007 USA; 4 Department of Medical and Molecular Genetics, Indiana University School of Medicine, Indianapolis, IN 46202 USA

**Keywords:** Cancer models, Ovarian cancer

## Abstract

Epithelial ovarian cancer is the most lethal gynecologic malignancy and one of the most common causes of cancer mortality among women worldwide. Ubiquitin-Specific Peptidase 13 (USP13) gene copy is strongly amplified in human epithelial ovarian cancer, and high USP13 expression is correlated with poor survival outcomes. Yet, its pathological contribution to ovarian tumorigenesis remains unknown. We crossed a conditional *Usp13* overexpressing knock-in mouse with a conditional knockout of *Trp53* and *Pten* mouse and generated a novel ovarian cancer genetically engineered mouse model (GEMM), which closely recapitulates the genetic changes driving ovarian cancer in humans. Overexpression of USP13 with deletion of *Trp53* and *Pten* in murine ovarian surface epithelium accelerated ovarian tumorigenesis and led to decreased survival in mice. Notably, USP13 greatly enhanced peritoneal metastasis of ovarian tumors with frequent development of hemorrhagic ascites. The primary and metastatic tumors exhibited morphology and clinical behavior similar to human high-grade serous ovarian cancer. Co-inhibition of USP13 and AKT significantly decreased the viability of the primary murine ovarian cancer cells isolated from the GEMM. USP13 also increased the tumorigenic and metastatic abilities of primary murine ovarian cancer cells in a syngeneic mouse study. These findings suggest a critical role of USP13 in ovarian cancer development and reveal USP13 as a potential therapeutic target for ovarian cancer.

## Introduction

Epithelial ovarian cancer (EOC) is the most deadly gynecological malignancy, with an estimated five-year survival rate of <29% for patients with advanced disease [1]. Accumulation of malignant ascites is a common feature of the advanced-stage ovarian cancer [2, 3]. High-grade serous ovarian cancer (HGSOC) is the most common and most lethal type of EOC, accounting for approximately 75% of ovarian cancers [4–7]. Except for *TP53* mutations, which are nearly ubiquitous, the recurrent mutations in oncogenes or tumor suppressor genes are relatively uncommon in HGSOC [8, 9]. Instead, HGSOC is characterized by a high rate of genomic alterations, with frequent copy number gains and losses [8–11]. For example, PI3K/AKT pathway is frequently activated in HGSOC via gene copy number alterations [10, 12]. In HGSOC, amplifications of the p110 subunit of PI3K (*PIK3CA*) have been described in 20% of cases, amplification of the AKT isoforms (*AKT1*, *AKT2*, or *AKT3*) occurs in 15 to 20%, while *PTEN* deletions have been described in 5% [9, 13].

Most EOC patients are diagnosed at an advanced stage, with widespread abdominal metastatic spread, high rates of recurrence, and chemoresistance [4, 5]. Overall survival rates for patients with EOC have not changed over the past 30 years [1]. Therefore, a better understanding of the risk factors and pathogenesis associated with this disease is critical to improve early detection, devise prevention strategies and develop more effective therapies [7, 14–16]. Genetically engineered mouse models (GEMMs) of ovarian cancer, involving genes expressed or deleted specifically in the ovary or the fallopian tube, allow us to evaluate the physiological relevance of defined genetic changes in ovarian cancer and to better understand the mechanistic significance of genetic lesions revealed in human tumor [17]. Ovarian cancer GEMMs, which can closely recapitulate human disease, have the potential to greatly improve our understanding of ovarian cancer progression and metastasis [17, 18].

Ubiquitin-Specific Peptidases (USPs), as deubiquitinating enzymes, catalyze the removal of ubiquitin moieties from their specific substrates [19, 20]. Among 50 USPs, only Ubiquitin-Specific Peptidase 13 (*USP13)* gene is strongly amplified in HGSOC [21]. *USP13* gene is localized in the amplicon across from chromosome 3q26.2–3q26.3, which is one of the most frequently amplified genomic loci in the HGSOC [9, 21]. USP13 controls the ubiquitination status of its multiple substrates involved in multiple biological processes, including cell cycle regulation, autophagy, metabolism, DNA repair response, and innate antiviral immunity [21–25]. Previously, we reported that USP13 is strongly overexpressed in HGSOC tissues, and the depletion of USP13 selectively kills USP13-amplified human ovarian cancer cells and suppresses ovarian tumor growth in a xenograft mouse study, suggesting a critical role for USP13 in the survival and proliferation of ovarian cancer cells [21]. Although the biochemical and molecular functions of USP13 have been explored in the various contexts of human cancer cells, including ovarian cancer, the in vivo pathological function of USP13 in cancer development and progression remains unknown.

Here, we developed a novel ovarian cancer GEMM, in which USP13 is overexpressed in ovarian surface epithelium (OSE) with the deletion of *Trp53* and *Pten* by ovarian intrabursal administration of Cre recombinant adenovirus. We found that USP13 overexpression promotes the development of highly aggressive epithelial serous ovarian tumors with rapid peritoneal metastasis and hemorrhagic ascites formation, which led to decreased survival in mice. The GEMMs, primary ovarian cancer cells, and a syngeneic study demonstrated that USP13 enhances tumorigenic and metastatic phenotypes of ovarian cancer. Inhibition of USP13 sensitized primary ovarian cancer cells to the effects of an AKT inhibitor. These findings suggest the important role of USP13 in ovarian tumorigenesis and metastasis and may lead to the development of new targeted therapies for ovarian cancer.

## Result

### *USP13* is highly amplified in human EOC and correlates with poor survival

**Fig. 1:**
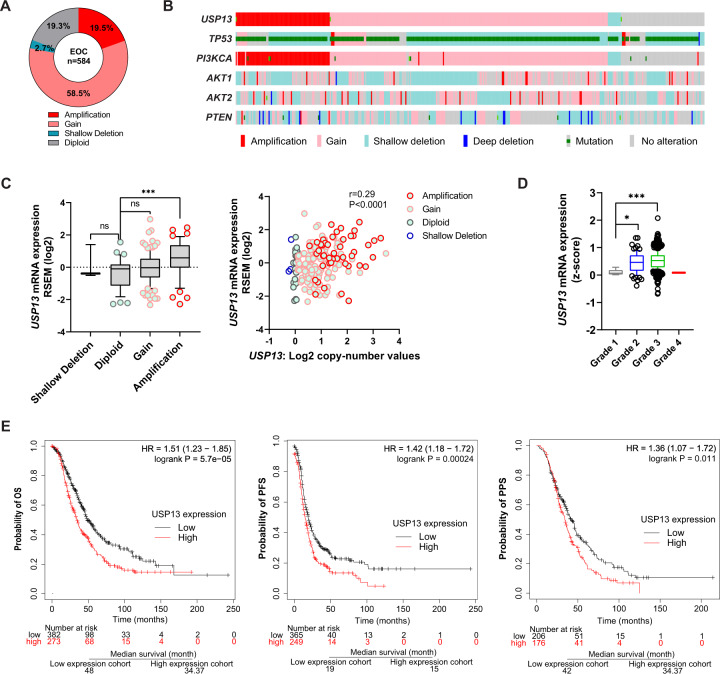
High USP13 expression correlates with poor survival in ovarian cancer patients. **A** The percentage of genomic alterations of *USP13* in human ovarian serous adenocarcinoma (TCGA provisional databases, cBioportal). **B** OncoPrint showing genetic alterations of *USP13*, *TP53*, *PI3KCA*, *AKT1*, *AKT2*, and *PTEN* in human ovarian serous adenocarcinoma. **C** Correlation between the copy number alteration and mRNA expression of *USP13* (left panel). Scatterplots of *USP13* copy number versus mRNA expression in ovarian serous adenocarcinoma (right panel). **D** *USP13* expression in different grades of ovarian cancer. **E** Kaplan–Meier plots of overall survival (OS), progression-free survival (PFS), and post-progression survival (PPS) for *USP13* expression in patients with ovarian cancer.

In normal human tissues, *USP13* mRNA expression is enriched in heart and skeletal muscle (Fig. S1A). Analysis of TCGA data indicates that *USP13* is rarely mutated, but the *USP13* gene is highly amplified (19.5% Amp) or gained (58.5 % Gain) in human ovarian serous cystadenocarcinoma (Fig. 1A). This genomic amplification of *USP13* has been identified in other human cancers, including esophageal squamous cell carcinoma (29.5% Amp), head and neck squamous cell carcinoma (15.1% Amp), endometrium carcinoma (8.8% Amp), and non-small cell lung cancer (18.1% Amp) (Fig. S1B). We found significant co-occurrence of *USP13* amplification with genetic alterations of *TP53*, *PI3KCA*, *AKT1*, *AKT2*, and *PTEN* in HGSOC (Fig. 1B). A high copy number of USP13 positively correlated with its mRNA expression in EOC and other cancers (Fig. 1C, S1C). *USP13* mRNA expression is increased in advanced ovarian tumors and correlates with tumor grade (Fig. 1D). In Kaplan–Meier plotter analysis [26], high *USP13* mRNA expression is significantly associated with decreases in overall survival (OS), progression-free survival (PFS), and post-progression survival (PPS) (Fig. 1E). Collectively, *USP13* is highly amplified in HGSOC, and it is associated with poor survival for ovarian cancer patients.

### Generation of ovarian surface epithelium (OSE)-specific USP13 overexpressing mouse models

**Fig. 2:**
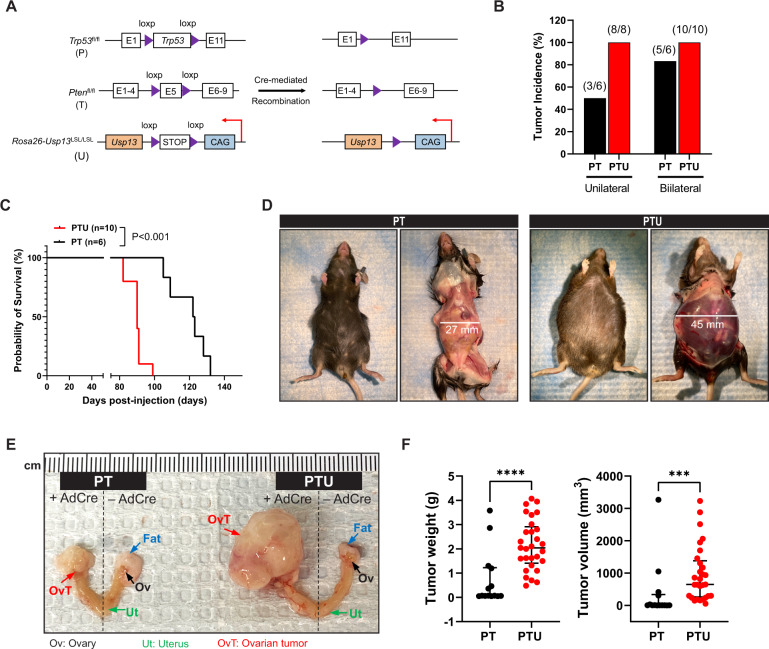
USP13 overexpression in OSE under *Trp53* and *Pten* depletion accelerates ovarian tumorigenesis and reduces survival rate. **A** Schematic depicting the *Trp53*^flox/flox^ (P), *Pten*^flox/flox^ (T), and *Usp13*^LSL/LSL^ (U) transgenes. Conditional overexpression of USP13 with deletion of *Trp53* and *Pten* by Cre-mediated recombination. **B** Ovarian tumor incidence of *Trp53*^flox/flox^; *Pten*^flox/flox^ (PT, *n* = 12) and *Trp53*^flox/flox^; *Pten*^flox/flox^; *Usp13*^LSL/LSL^ (PTU, *n* = 18) mice after unilateral or bilateral ovarian AdCre intrabursal injection. **C** Kaplan–Meier curve showing overall survival of PT (*n* = 6) or PTU (*n* = 10) mice with bilateral AdCre intrabursal injection. *P* < 0.001, log-rank test. **D** Representative image of PT and PTU mouse after 104 days of AdCre intrabursal bilateral injection. **E** Ovarian tumor development in PT and PTU mice. Left side is AdCre virus injected and right side is non-injected ovary. Ov, ovary; Ut, uterus; OvT, Ovarian tumor. **F** Primary tumor weight (left) and tumor volume (right) were measured in each ovary of all PT and PTU mice. Unpaired Student *t-test* was used for statistical analyses. ***P < 0.001.

To investigate the in vivo role of USP13 in ovarian cancer, we generated USP13 conditional overexpressing ovarian cancer GEMMs. First, we developed a conditional USP13 knock-in (KI) mouse model by inserting the LoxP-Stop-LoxP-*Usp13* allele with a ubiquitous CAG promoter into a ubiquitously expressed Rosa26 locus in C57BL/6J background (named as *Usp13*^*LSL*^, U) (Figs. 2A; S2A). In the *Usp13*^*LSL*^ mouse, Cre-mediated excision of the stop signal flanked by loxP sites allows for the specific overexpression of *Usp13*. Genotypes of founder mice were confirmed by genomic DNA PCR and southern blotting (Fig. S2B, C). Next, *Usp13*^*LSL*^ (U) was further crossed with *Trp53*^*flox/flox*^ mouse (P) and *Pten*^*flox/*flox^ mouse (T) (C57BL/6J, Jackson Laboratory) to generate the *Trp53*^*flox/flox*^; *Usp13*^*LSL/LSL*^ (PU) and *Trp53*^*flox/flox*^; *Pten*^*flox/flox*^; *Usp13*^*LSL/LSL*^ (PTU) mouse models (Fig. 2A). *Trp53*^*flox/flox*^ (P) mouse and *Trp53*^*flox/flox*^*; Pten*^*flox/flox*^ (PT) mouse, which does not overexpress USP13, were used as control groups in this study. We applied ovarian intrabursal administration of the Cre recombinant adenovirus (AdCre) to induce ovarian surface epithelium (OSE)-specific Cre-mediated recombination. We confirmed that the ovarian intrabursal AdCre infection successfully induces Cre-mediated genetic recombination in murine OSE. The expression of Cre recombinase and Cre-mediated loxP excision were specific to the ovarian epithelium, as no Cre and Cre-mediated excision were found in the liver, oviduct, and uterus (Fig. S2D). We were able to detect a hyperplasic change in the OSE of PT mice after 10 days of intrabursal AdCre injection, while no abnormality was observed in the other side of the control ovary from the same PT mice (Fig. S2E).

### Overexpression of *Usp13* accelerates ovarian tumorigenesis and causes poor outcomes in *Trp53*^*flox/flox*^; *Pten*^*flox/flox*^; *Usp13*^*LSL/LSL*^ (PTU) mice

To further determine how USP13 influences ovarian cancer initiation and progression, ovarian intrabursal AdCre injection was performed in 8–10 weeks old female PT and PTU mice. The total number of PT and PTU mice receiving AdCre unilateral (one ovary) or bilateral (both ovaries) injection, and their corresponding tumor incidences, are summarized in Table 1. AdCre administration into both ovaries (bilateral) increased the incidence of primary ovarian tumor formation and metastatic tumor development in both PT and PTU mice (Tables 1, 2). We found that although PT mice develop ovarian tumors, PTU mice showed a significantly higher tumor incidence (100% by unilateral and 100% by bilateral) compared to PT mice (50% by unilateral and 83.3% by bilateral) (Table 1; Fig. 2B). At 69 days after AdeCre injection (unilateral), PTU mice already developed ovarian tumor formation, but not in PT mice, suggesting faster tumor initiation in PTU mice (Fig. S3A). We determined the survival of PT and PTU mice with bilateral AdCre intrabursal injection. PTU mice showed worse survival rates compared to PT mice (Fig. 2C). The PT group had a median survival of 122.5 ± 4.3 days, while the median survival for the PTU group was 90 ± 1.5 days (Fig. 2C). Next, we measured tumor burden in PT vs. PTU mice. At 104 days post-unilateral injection of AdCre, PTU mice showed peritoneal cavities filled with hemorrhagic ascitic fluid, while PT mice exhibited no ascites accumulation (Fig. 2D). Examination of the reproductive tracts of PT and PTU mice, revealed that PTU mice developed massive ovarian tumors compared with PT mice (Fig. 2E). Tumor weight (2.9-fold) and volume (2.7-fold) were substantially increased in PTU mice (Fig. 2F). In addition, notable hyperplasia was detected in OSE of 60% of PU (*n* = 5) mice, but not in P (*n* = 4) mice at 109 post-AdCre intrabursal unilateral injection (Fig. S3B). Collectively, in the context of p53 deletion, OSE-specific overexpression of USP13 is able to induce hyperplasia in OSE of PU mice, and additional deletion of PTEN is important to drive ovarian tumor formation in PTU mice. Compared to PT mouse, USP13 overexpression significantly accelerated ovarian tumor development and progression and led to worse survival in PTU mice within the context of p53 and PTEN inactivation.

**Table 1:** Summary of ovarian tumor incidence following intrabursal injection of AdCre virus.

Genotype	Number of mice evaluated	Number of mice develop ovarian tumor (%)	Mean survival (range)
*Trp53*^*flox*/*flox*^; *Pten*^*flox*/*flox*^ (PT), *n* = 12			
Unilateral	6	3 (50)	
Bilateral	6	5 (83.3)	122.5 ± 4.3 days (105–132)
*Trp53*^*flox*/*flox*^; *Pten*^*flox*/*flox*^; *Usp13*^*LSL*/*LSL*^ (PTU), *n* = 18			
Unilateral	8	8 (100)	
Bilateral	10	10 (100)	90 ± 1.5 days (82–99)
WT (Bilateral)	4	0	

*Unilateral* AdCre injection only into right ovary; *Bilateral* AdCre injection into both ovaries.

**Table 2:** Summary of metastatic tumor incidence following intrabursal injection of AdCre virus.

Genotype	Number of mice evaluated	Peritoneal tumor (%)	Mesenteric tumor (%)	Spleen (%)	Diaphragm (%)	Ascites (%)
*Trp53*^*flox*/*flox*^; *Pten*^*flox*/*flox*^ (PT), *n* = 12						
Unilateral	6	0	0	0	0	0
Bilateral	6	1 (16.6)	0	0	2 (33.3)	2 (33.3)
*Trp53*^*flox*/*flox*^; *Pten*^*flox*/*flox*^; *Usp13*^*LSL*/*LSL*^ (PTU), *n* = 18						
Unilateral	8	1 (12.5)	0	0	1 (12.5)	1 (12.5)
Bilateral	10	7 (70)	7 (70)	7 (70)	7 (70)	8 (80)

*Unilateral* AdCre injection only into right ovary; *Bilateral* AdCre injection into both ovaries.

### USP13 overexpression promotes metastasis of ovarian tumors in PTU mouse models

**Fig. 3:**
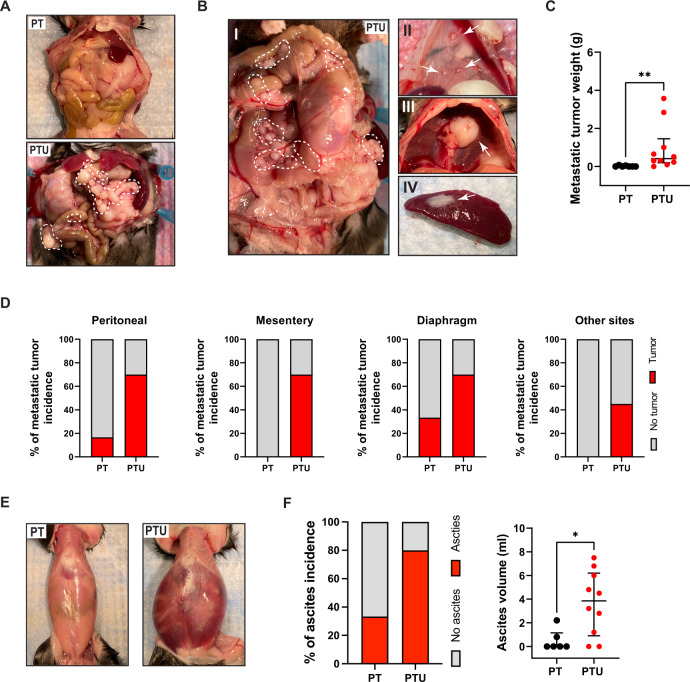
USP13 overexpression promotes metastasis of ovarian cancer. **A** Representative gross anatomy of PT (*n* = 6) and PTU (*n* = 10) mice after 104 days AdCre bilateral bursal injection. PTU mice showed widespread large metastases (white dotted line) in peritoneal cavity. **B** Exposed abdominal cavity of PTU mouse, showing metastasized tumor mass in mesentery (I), peritoneal wall (II), diaphragm (III), and spleen (IV). Mesentery tumor nodules (dotted line) and metastatic tumors (arrow). **C** Weight of metastatic tumors of PT and PTU mice. **D** Incidence (%) of metastasis by body sites in PT and PTU mice. **E** PTU mouse showing abdominal distension and hemorrhagic ascitic fluid. **F** Incidence of presence of ascites (left) and ascites volume (right) in PT and PTU mice. Unpaired Student *t-test* was used for statistical analyses. *P < 0.05, **P < 0.01.

Next, we assessed metastatic phenotypes of ovarian tumors in PT and PTU mice. The metastatic tumor incidence and ascites formation for each group are summarized in Table 2. In addition to the massive primary ovarian tumor, PTU mice developed aggressive peritoneal spread of metastatic tumors, while metastatic tumors were barely observed in PT mice (Fig. 3A; Table 2). PTU mice showed frequent metastatic tumor development in metastatic sites (omentum, mesentery, peritoneal wall, pelvic, and diaphragm) common in human HGSOC (Fig. 3B; Table 2). The weights of metastatic tumors of PTU mice were significantly higher than in PT mice (Fig. 3C). Compared to PT mice with bilateral AdCre injection, PTU mice exhibited significantly enhanced tumor metastasis to peritoneal wall (16.6% vs. 70%), mesentery (0% vs. 70%), diaphragm (33.3% vs. 70%), or other distant sites (pelvic, kidney, and spleen; 0% vs. 70%) (Fig. 3D). In particular, 80% of the PTU mice group (8/10 mice, by bilateral AdCre injection) developed hemorrhagic ascitic fluid (Fig. 3E; Table 2). Ascites incidence and ascites volume were significantly increased in PTU mice (Fig. 3F; Table 2). These results suggest that USP13 overexpression not only accelerates ovarian tumor development but also promotes the metastatic ability of these ovarian tumors, while conditional inactivation of *Pten* and *Trp53* in OSE is not sufficient for the development of metastatic ovarian cancer.

### Characterization of OSE-derived PTU primary and metastatic tumors

**Fig. 4:**
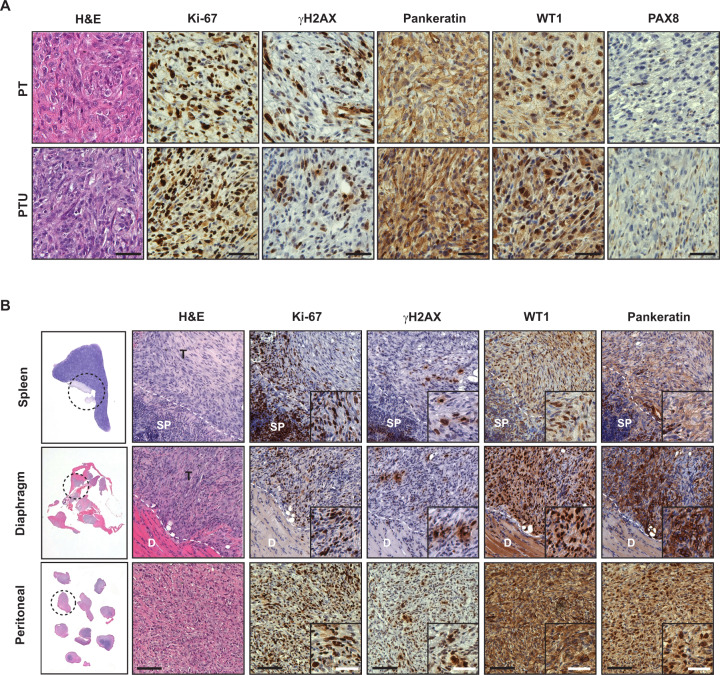
Characterization of primary and metastatic ovarian tumors from PT and PTU mice. **A** Histopathological (H&E stain) and IHC analysis (Ki-67, γ-H2AX, Pankeratin, WT1, and PAX8) of ovarian tumors isolated from PT and PTU mice. Scale bars = 50 μm. **B** H&E stain and IHC analysis (Ki-67, γ-H2AX, WT1, and Pankeratin) of metastatic tumors from spleen, diaphragm, and peritoneal of PTU mice. White dotted line indicates borderline between original tissue and tumor. Black scale bars = 100 μm, white scale bars = 50 μm.

Primary and metastatic tumors were dissected from PT and PTU mice at the end-point, and the histopathological features were examined using immunohistochemical (IHC) analyses. USP13 overexpression in PTU tumors was confirmed by immunoblotting (Fig. S3C). PT and PTU tumors revealed high-grade serous histology with tumor cells of nuclear atypia, pleomorphic nuclei, and abnormal mitotic figures (Fig. 4A, S3D). There was no obvious difference in the microscopic appearance between PT and PTU tumors. The primary ovarian tumors that arise in the setting of PTU have similar histological features to PT mice. Both PT and PTU tumors were highly positive for Ki-67 (proliferation marker) and γH2AX (DNA damage marker), indicating that tumors are highly proliferative (Fig. 4A). Wilms’ tumor 1 (WT1) was positive in PT and PTU tumors (Fig. 4A). Epithelial markers such as Pankeratin and CK7 were positive in PT and PTU tumors, indicating epithelial tumors (Fig. 4A, S3E). PAX8 (fallopian originated marker) and Inhibin α (granulosa marker) were not detected in PT and PTU tumors (Fig. 4A, S3E). These data suggest that PT and PTU mice develop epithelial tumors, which are originated from the ovarian epithelium but not from granulosa cells within the ovaries or fallopian tubes. Next, we assessed metastases isolated from PTU mice, including metastatic tumors to spleen, diaphragm, and the peritoneal wall. Infiltration of tumors was observed in H&E stain (Fig. 4B). Similar to the primary tumors, PTU metastatic tumors were positive for Ki-67, γH2AX, WT1, and Pan-Keratin (Fig. 4B). Collectively, OSE-derived primary and metastatic ovarian tumors in PTU mice displayed characteristics similar to those of human HGSOC.

### Invasive, metastatic, and tumorigenic properties are enhanced in PTU primary cancer cells

**Fig. 5:**
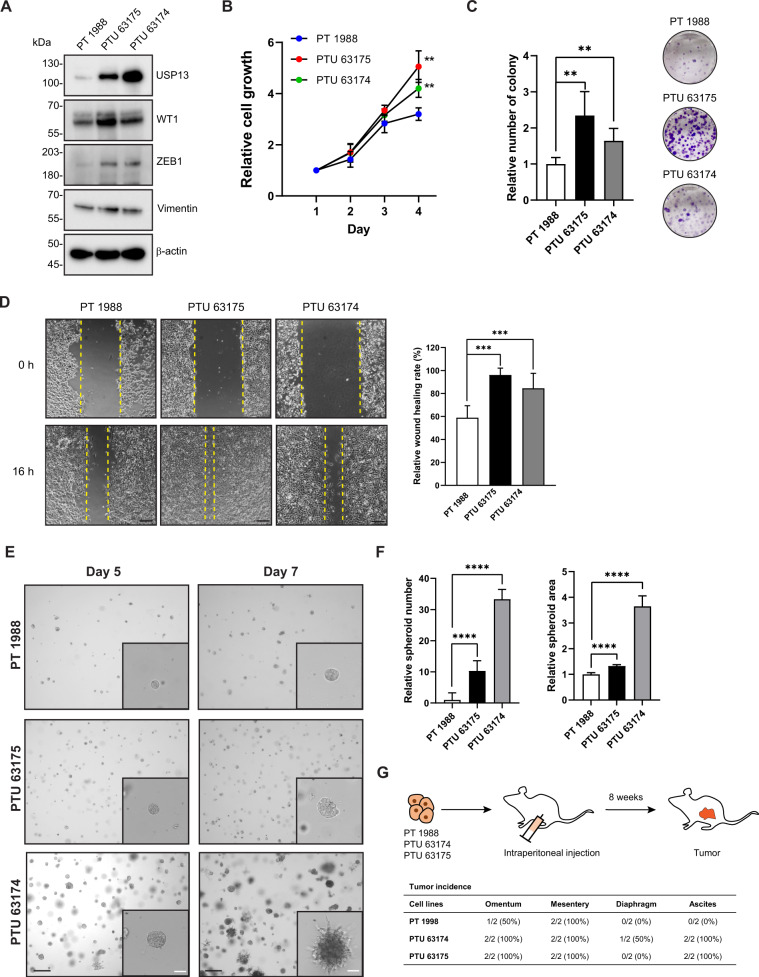
Invasive, metastatic, and tumorigenic properties are enhanced in PTU primary cancer cells. **A** Western blot analysis of USP13, WT1, ZEB1, and Vimentin expression in ovarian primary cancer cell lines established from PT and PTU mice. **B** Cell proliferation of PT and PTU primary cancer cells was measured by MTS assay. Data are presented as the mean of three independent experiments. Error bars denote the standard deviation (**P < 0.01 vs PT). **C** Clonogenic assay of PT and PTU cells in anchorage-dependent culture for 7 days and colonies were stained by 0.05% crystal violet. Representative images are shown (right). Unpaired Student *t-test* was used for statistical analyses. **D** Wound-healing assay of PT and PTU cell lines. Experiments were performed in triplicate and representative images (left) are shown. The average wound area was calculated using Image J. Graph (right) showing relative wound healing rate to 0 h (means ± SD) of PT and PTU cells in three independent experiments. ***P < 0.001, two-tailed Student *t-test*. **E** Tumor spheroid culture of PT and PTU cell lines in Matrigel domes for 5 or 7 days, and imaged by brightfield microscopy. Black scale bars = 250 μm, white scale bars = 50 μm. **F** Relative mean number of spheroids ± SD (*n* = 3, >6 fields/ cell line, Student *t-test*) (left) or relative mean of spheroid area ± SD (*n* = 3, >52 spheroid/ cell line, Student *t-test*) (right). (**G**) Schematic of intraperitoneal injection of 5 × 10^5^ PT or PTU cells into 7 weeks old female wild-type C57BL/6J mouse. Tumor and ascites incidence were evaluated 8 weeks post-injection and results are summarized in the table below.

To further characterize the cancer cells of PT and PTU ovarian tumors, we established cancer cell lines from the primary ovarian tumors of one PT mouse (#PT 1988) and two PTU mice (#PTU 63174, #PTU 63175). While PT 1988 cells expressed low USP13, both PTU cell lines (PTU 63174, PTU 63175) expressed a high level of USP13 (Fig. 5A). PTU 63174 cells had higher USP13 expression than PTU 63175. All cell lines were positive for WT1 (Fig. 5A). ZEB1 promotes epithelial-mesenchymal transition (EMT), cancer cell plasticity, dissemination, and drug resistance [27–29]. Interestingly, ZEB1 expression was upregulated in both PTU primary cell lines (Fig. 5A), suggesting it may act as a factor promoting the metastatic spread of the PTU tumors. Both PTU cell lines showed increased cell proliferation compared to PT cells (Fig. 5B) and higher colony formation ability under attachment-dependent culture conditions (Fig. 5C). PTU cell lines also demonstrated enhanced migration in a wound-healing assay (Fig. 5D).

Ovarian cancer metastasizes largely within the peritoneal cavity via transcoelomic metastasis, where cells disseminate as single cells or clusters into the ascites and metastasize to the peritoneum and omentum through peritoneal fluid [30, 31]. The survival of cancer cells in anchorage-independence is an essential step in transcoelomic metastasis, which is marked by an initial resistance to anoikis, adaptations, and long-term survival, including the formation of spheroids in the ascites [32, 33]. We assessed PT and PTU cancer cell growth in three-dimensional (3D) culture systems. The same number of PT and PTU cells were embedded in a Matrigel dome or seeded in culture media for an attachment-independent spheroid culture condition. In a 3D Matrigel dome culture, both PTU 63174 and PTU 63175 cells formed a greater number of spheroids than PT 1988 cells. The PTU spheroids also grew larger than PT 1988 spheroids (Fig. 5E, F). However, the spheroid size of PTU 63174, the cell line with the highest USP13 expression, was dramatically larger than others (Fig. 5E, F). Interestingly, some of the PTU spheroids exhibited a protrusion phenotype (Fig. 5E, S4A). Similarly, an increased spheroids number and invasive phenotypes of PTU spheroids were found in a low-attachment media culture condition with addition of 2% Matrigel (Fig. S4B).

Next, we further examined the tumorigenic and metastatic characteristics of PTU cells using an in vivo syngeneic study. 5 × 10^5^ PT or PTU cells were intraperitoneally (IP) injected into 7 weeks old C57BL/6 wild-type female mice, respectively. At 8 weeks post-IP injection, both PTU cell lines (PTU 63174 and PTU 63175) developed large tumors throughout the peritoneal cavity with 100% ascites formation, whereas IP injection of PT 1988 cells led to the development of very small tumors with no ascites development (Figs. 5G, S4C). Collectively, PTU spheroids showed increased spheroid formation ability with invasive and disseminated phenotypes, and USP13 expression level seems to correlate with these phenotypes. Moreover, robust tumorigenic and metastatic features of PTU cell lines were preserved in a syngeneic animal study. These data from 3D culture conditions and a syngeneic in vivo mouse study support that USP13 not only plays a role in ovarian tumorigenesis but also is critical for ovarian cancer cell invasion and metastasis.

### USP13 overexpression affects cancer cell response to chemotherapeutics and ATK inhibitor

**Fig. 6:**
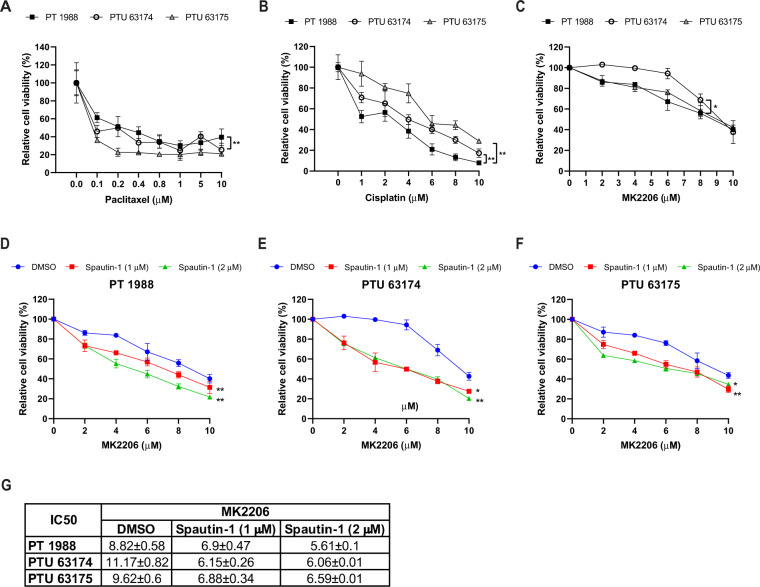
Inhibition of USP13 sensitizes primary ovarian cancer cells to the AKT inhibitor MK2206. Response of PT and PTU primary ovarian cancer cells to chemotherapy **A** Paclitaxel (48 h, 0–1 μM), **B** Cisplatin (48 h, 0–10 μM), or **C** AKT inhibitor MK-2206 (24 h, 0–10 μM). Cell viability was determined by MTT assay and calculated the relative cell viability to the vehicle (DMSO) only treated cells. Error bars, mean ± SD, *n* = 3. **D–F** PT and PTU-derived cells were treated with a combination of Spautin-1 (1 or 2 μM), a USP13 inhibitor, and MK-2206 for 24 h. **G** The IC50 values were summarized. Error bars, mean ± SD, with *n* = 3. *P < 0.05, **P < 0.01, ****P < 0.0001 (paired Student’s t-test).

HGSOC are known to be highly responsive to initial chemotherapy such as paclitaxel or cisplatin, which are first-line treatments for the advanced ovarian cancer [34, 35]. PT and PTU cancer cells were treated with paclitaxel or cisplatin, and cell viability was measured using an MTT assay. PTU cells (PTU 63175) were more sensitive to paclitaxel compared to PT cells (Fig. 6A). In contrast, PTU 63174 and PTU 63175 cells were more resistant to cisplatin treatment compared to PT cells (Fig. 6B). The PI3K/AKT/mTOR pathway is frequently activated in HGSOC [10, 12]. Co-occurrence of *USP13* amplification and PI3K/AKT pathway alteration has been found in HGSOC (Fig. 1B). Therefore, we examined a combinatory effect of ATK inhibition and USP13 inhibition on USP13-overexpressing PTU cancer cells. Interestingly, PTU 63714 cells were most resistant to the pan-AKT inhibitor MK-2206 (Fig. 6C). PTU 63174 cells (IC50, 11.17 μM) and PTU 63175 (IC50, 9.62 μM) were relatively more resistant to MK-2206 treatment than PT cells (IC50, 8.82 μM). Compared to a single treatment of MK-2206, co-treatment of Spautin-1 and MK-2206 decreased cell viability of both PT and PTU cells (Fig. 6D–F).

## Discussion

In the present study, we developed a conditional overexpressing USP13 knock-in (*Usp13*^*LSL*^) mouse model. We combined this mouse model with *Trp53*^*flox*^ and *Pten*^*flox*^ genetic background to investigate the function of USP13 in ovarian cancer development. This novel ovarian cancer GEMM recapitulates the clinical feature of human HGSOC. The ability of USP13 to enhance tumorigenesis and metastasis, to our knowledge, has not been previously reported. OSE-derived USP13 overexpression promoted the development of ovarian tumors, which are similar to that observed in women, with several characteristics of HGSOC, including poorly differentiated serous histology, peritoneal metastasis, development of malignant ascites, and enhanced sensitivity to cisplatin and paclitaxel. OSE-derived PTU tumors also revealed significantly enhanced metastatic spread throughout the peritoneal cavity with frequent development of hemorrhagic ascites.

The molecular mechanism of USP13 in hyperplastic transformation of murine OSE is currently unclear. Also, it is not clear whether or how the USP13, p53, and PTEN signaling cascades work together to promote transformation and metastasis in this PTU ovarian cancer GEMM. Previous studies on USP13 may suggest potential molecular mechanisms by which USP13 promotes ovarian tumorigenesis or progression. For example, USP13 is involved in inducing Beclin-1-mediated autophagy [22]. Although autophagy prevents tumorigenesis in normal cells, it can promote the survival of cancer cells by providing essential building blocks for cell growth, cancer cell stemness, and cell migration and invasion [36–41]. Therefore, USP13-mediated Beclin-1 stabilization could promote autophagy of ovarian cancer under metabolic stress conditions, and it could be one of the possible mechanisms for the enhanced ovarian tumor metastasis in PTU mice. USP13 can regulate microphthalmia-associated transcription factor (MITF), an essential modulator of melanoma growth [42]. USP13 also functions as an oncogene by stabilizing MCL1 apoptosis regulator (MCL1) in lung and ovarian cancer cells [25]. USP13 also regulates the RAP80-BRCA1 complex dependent DNA damage response by deubiquitinating Receptor-associated protein 80 (RAP80) [24], and is negatively involved in innate immunity by stabilizing Stimulator of interferon genes (STING) [23]. However, USP13 also appears to stabilize PTEN, implicating a tumor-suppressive role for USP13, at least in breast cancer [43]. In our previous study, we identified that USP13 increased both energy production and biosynthesis in human ovarian cancer cells by stabilizing Oxoglutarate Dehydrogenase (OGDH) and ATP Citrate Lyase (ACLY), which are key metabolic enzymes driving the reprogramming of ovarian cancer cell metabolism [21]. The contributions of *PTEN* and *TP53* dysregulation have been reported in human ovarian cancer and mouse models [44–47]. Previous animal studies also show that PTEN loss accelerates tumor development in related HGSOC GEMMs [48, 49]. Previously, knockdown of USP13 sensitized USP13-overexpressing human ovarian cancer cells to the treatment of AKT inhibitor [21]. Similarly, inhibition of USP13 sensitized primary PTU cancer cells to the effects of an AKT inhibitor (Fig. 6). This finding suggests that USP13 and PI3K/AKT may share a downstream pathway(s), which are important for ovarian cancer cell survival as well as tumorigenesis. USP13 amplification in human HGSOC may contribute to the development of resistance of PI3K/AKT inhibitors in ovarian cancer, and targeting USP13 may overcome cancer cell resistance against targeted therapy for PI3K/AKT/mTOR pathway. Interestingly, PT and PTU cells respond to cisplatin and paclitaxel differently. One possible explanation is that chemo drugs could preferentially kill cells with higher proliferation rates. Indeed, PTU cells demonstrated a higher proliferation rate compared to PT cells. Therefore, the proliferation rate of our primary cancer cells might affect the sensitivity of cancer cells to chemotherapy. In addition, cancer cell metabolism or some other mechanism driven by USP13 that would increase sensitivity to paclitaxel. Currently, we do not know the molecular mechanism of how USP13 is related to chemosensitivity. USP13 enhanced metastasis and ascites development in GEMM (Fig. 3) and increased cancer cell migration, 3D-spheroid forming efficiency, and tumorigenesis in syngeneic mice (Fig. 5). Therefore, it may be possible that USP13 overexpression could increase cancer stemness, which also could increase cisplatin resistance. Future studies will focus on proteomics, metabolomics, and molecular mechanistic understanding of how USP13 promotes tumorigenesis and metastatic features of ovarian cancer.

Extensive molecular and histological characterizations of human tissues have suggested various cell-of-origin of HGSOC [50–54]. Historically, the ovarian surface epithelium (OSE) and, more recently, the fallopian tube epithelium (FTE) are considered as compelling origins for HGSOC [55–58]. Several ovarian cancer GEMM studies have clearly demonstrated that the cell of origin and genetics play a crucial role in determining tumor phenotype [49, 57]. Cell of origin also appears to have profound effects on the phenotype of tumors induced by the same genetic defects [59, 60]. Recent studies show that the cell of origin significantly affects not only HGSOC growth and metastasis, but also drug response [52, 61–63]. Our GEMMs, described here, are of OSE origin. We used ovarian bursa AdCre injection, which has been widely used for the introduction of genetic alterations in OSE of GEMMs due to the lack of OSE-specific promoters [17]. Notably, FTE is considered as a strong potential cell of origin for HGSOC [49, 51, 60, 64]. Therefore, it will be important to investigate the impact of USP13 on FTE-derived ovarian tumorigenesis. Our *Usp13*^*LSL*^ mouse model provides a powerful tool for elucidating the effect of USP13 when combined with various tissue-specific Cre driver lines. The combination of *Usp13*^*LSL*^ with FTE-specific Cre mice lines (*Ovgp1*-iCreER^T2^ [49] or *Pax8rtTA* [48]) could be used to investigate the function of USP13 in FTE-origin HGSOC as well as differences in drug responsiveness of tumors with USP13 gene overexpression from OSE vs. FTE cell of origin. Furthermore, our model is generated from a pure C57BL/6J background; thus, the primary ovarian cancer cells can be used in syngeneic mouse studies. Therefore, our primary cancer cells will be useful tools for the investigation of an ovarian tumor-host interface, especially the tumor-immune microenvironment.

In conclusion, PTU ovarian tumor mouse model described here has genetic defects, morphology, and clinical behavior similar to human HGSOC. Such preclinical models, including primary ovarian cancer cell lines available for a syngeneic mouse study, should prove to be useful tools in evaluating chemotherapeutic and targeted therapy strategies for ovarian cancers.

## Materials and methods

### Generation of conditional USP13 knock-in mouse (*Usp13*^*LSL*/*LSL*^, U) and *Trp53*^*flox*/*flox*^; *Pten*^*flox*/*flox*^; *Usp13*^*LSL*/*LSL*^ mouse (PTU) models

All animal experiments were performed in accordance with protocols approved by the Institutional Animal Cancer and Use Committees of the Georgetown University. Conditional USP13 knock-in (KI) mouse model (named as *Usp13*^*LSL*^, U) was generated by inserting the LoxP-Stop-LoxP-*Usp13* allele with a ubiquitous CAG promoter into a ubiquitously expressed Rosa26 locus, through injection of mouse *Usp13* cDNA and Cas9 mRNA into the pronuclei of fertilized eggs (C57BL/6J strain) by Cyagen (Santa Clara, CA). For genotyping of USP13 knock-in mice, genomic DNA was isolated from tail clippings using the Cyagen Biotech (Los Angeles, CA) according to the manufacturer’s protocol. PCR was performed using 10–100 ng of genomic DNA as template and the following transgene specific primers: wild-type forward, 5′-CACTTGCTCTCCCAAAGTCGCTC-3′ flox forward, 5′- AGATGT ACTGCCAAGTAGGAAAGTC-3′ reverse, 5′-ATACTCCGAGGCG GATCACAA-3′ (wild-type, 453 bp; flox, 616 bp). Thermal cycling conditions using master mix (Takara, Mountain View, CA) were as follows: initial denaturation at 95 °C for 5 min, followed by 95 °C for 30 s, 60 °C for 35 s, 72 °C for 35 s, for a total of 33 cycles, followed by a final extension at 72 °C for 5 min. C57BL/6J *Trp53*^*flox*/*flox*^ (P) mouse and C57BL/6J *Pten*^*flox*/*flox*^ (T) mouse were purchased from The Jackson Laboratory (#008462, #006440). *Trp53*^*flox*/*flox*^; *Pten*^*flox*/*flox*^; *Usp13*^*LSL*/*LSL*^ (PTU) mouse was established by breeding *Usp13*^*LSL*/*LSL*^ (U) with *Trp53*^*flox*/*flox*^*;Pten*^*flox*/*flox*^ (PT) mice.

### Intrabursal injection of adenovirus expressing Cre recombinase

Replication-incompetent recombinant adenovirus expressing Cre recombinase under the control of CMV promoter (Ad5-CMV-Cre, AdCre) [65] was obtained from UI viral vector core, University of Iowa (Iowa City, IA). Ovarian bursa injection of AdCre was performed as described previously [66, 67]. The ovaries were accessed and exposed through a dorsal incision and 5 × 10^7^ plaque-forming units (pfu) of AdCre in a total volume of 3 μl was injected with a 34-gauge needle coupled with a Hamilton syringe into the right side of one ovary (unilateral) or both ovaries (bilateral) bursal cavity of 8 to 10 weeks old female mice. A total of 12 PT, 18 PTU, and 4 (control) C57BL6 wild-type mice underwent intrabursal injection of AdCre. Localization of Cre expression to the ovarian surface epithelium after intrabursal injection of AdCre was evaluated using genomic DNA PCR. A week after a single intrabursal injection of AdCre into a PT mouse, the mouse was euthanized and liver, ovary, oviduct, and uterus were harvested, and genomic DNA was isolated using DirectPCR Lysis Reagents (Viagen). Lysate of OSE was enriched by limiting the initial digestion time of ovary specimens at 55 °C to 1 h. After the first 1 h, the sample was centrifuged briefly at low speed (1000 rpm) to pellet the undigested ovary. The supernatant was collected, and genomic DNA was immediately isolated. The pellet was resuspended and digested overnight (12 h) along with other tissues. For detection of Cre recombinase and Cre-mediated excision of *Trp53*^*flox*/*flox*^ and *Pten*^*flox*/*flox*^ allele, specific primer sets were used (Supplementary Table 1).

### Histology and Immunohistochemistry

The tissues and tumors were removed, fixed, and paraffin-embedded. Five μm serial sections were cut for Hematoxylin and Eosin stains (H&E) and immunohistochemistry (IHC) analysis. Paraffin sections were deparaffinized in xylene and rehydrated in ethanol according to standard protocol. High-temperature antigen retrieval was performed using sodium citrate buffer (Vector laboratories, Burlingame, CA), and endogenous peroxidase activity was blocked using 3% hydrogen peroxide (Sigma, St. Louis, MO). All samples were blocked using an avidin/biotin blocking kit (Vector laboratories). Primary antibodies were diluted in TBS-T (0,05% Tween-20) at the following concentrations: Ki-67 (1:500, Cell signaling technology), γH2AX (1:600, Cell signaling technology, Danvers, MA), Pan-keratin (1:2000, Proteintech, Rosemont, IL), CK7 (1:400, Proteintech), PAX8 (1:600, Proteintech), and Inhibin α (1:500, Proteintech). After washing, sections were incubated with biotinylated goat anti-rabbit IgG (Vector laboratories). Sections were incubated with VECTASTAIN ABC Reagent (Vector laboratories) and then developed using a DAB peroxidase substrate kit (Vector laboratories). Counterstaining was performed using hematoxylin (Leica, Buffalo Grove, IL).

### Western blot

Tumors and cells were washed with ice-cold phosphate-buffered saline and lysis with NP-40 lysis buffer containing protease inhibitors and phosphatase inhibitors (Thermo Fisher). Protein concentration was measured by Bio-rad protein assay dye reagent concentrate. Protein samples were subjected to SDS-PAGE and transferred to polyvinylidene difluoride membranes (Bio-rad, Hercules, CA). The membranes were incubated overnight at 4 °C with primary antibodies: USP13 (1:500, Santa Cruz Biotechnology), WT1 (1:1000, Proteintech), ZEB1 (1:1000, Cell signaling technology), Vimentin (1:1000, Cell signaling technology), and β-actin (1:500, Santa Cruz Biotechnology, Dallas, Texas). After several washes, the membranes were incubated with secondary antibodies for 1 h at room temperature and developed with the Clarity Western ECL Substrate (Bio-rad). Membrane images were captured by ChemiDoc MP system (Bio-rad).

### Primary cell lines and Cell culture

Tumor-bearing mice were sacrificed, and primary ovarian tumors were excised. Tumors were briefly washed in ice-cold 1X D-PBS and transferred to 1X D-PBS containing dishes. Tumor mass was minced and incubated with DMEM culture media containing 1 mg/ml Collagenase/Dispase (Sigma). Digested tissues were centrifuged and resuspended in DMEM (Thermo Fisher) containing 10% FBS (Sigma) and Pen/Strep (Thermo Fisher), and filtered with 100 μm mesh (Thermo Fisher). Cells were washed three times in 1 ml culture media and plated in a 6-well tissue culture dish. During subsequent plating, homogenous tumor cells were selected and subjected to further characterization and spheroid culture. Cell proliferation was measured using CellTiter 96® AQueous One Solution Reagent (Promega, WI).

### Drug treatment and Cytotoxic assays

Cisplatin, Paclitaxel, Spautin-1, and MK-2206 were obtained from Selleck Chemicals (Selleckchem, Houston, TX). PT and PTU cells were seeded, and 24 h later, the cells were treated with Cisplatin, Paclitaxel, MK-2206, or combination treatments of Spautin-1 and MK-2206. After 24 h (for Spautin-1, MK-2206) and 48 h (for Cisplatin, Paclitaxel), cell viability was determined by the 3-(4,5-dimethylthiazol-2-yl)-2,5-diphenyltetrazolium bromide (MTT) assay (Sigma), and absorbance was measured by a plate reader at 570 nm. Data are represented as the mean ± standard deviation. Student t-tests were performed to compare cell viability across cell lines.

### Wound healing assay

PT and PTU cells were cultured as confluent monolayer in 12-well plates and carefully scratched with a 10 μL pipette tip. After washing with PBS to remove detached cells, images were captured at 16 h by microscope using 5x objectives. The area of the wound was analyzed using ImageJ.

### 3D Culture and Sphere formation assay

For sphere formation of PT and PTU cells, cells were plated at a density of 20,000 cells/well in a 24-well ultra-low attachment plate (Corning, Corning, NY) in DMEM containing 2% Matrigel (growth factor–reduced, phenol red-free; Corning) and 10% FBS (Thermo Fisher) for 5–7 days at 37 °C in humidified air containing 5% CO_2_. For the single-cell-derived sphere-forming assay under Matrigel dome-embedding conditions, 5,000 PT or PTU cells were suspended in 50 µl Matrigel and were carefully dispensed as droplets into a pre-warmed 24-well ultra-low attachment plate. After the Matrigel matrix dome was solidified in a humidified incubator at 37 °C for 15 min, 1 ml of warm DMEM with 10% FBS was added and cells were cultured for 7–10 days. The culture medium was changed every 2–3 days. Cells were observed daily, and spheres were counted under an inverted microscope (Leica Microsystems). To calculate the forming efficiency, spheres with diameters >50 µm were scored under an inverted microscope (Leica Microsystems). The sphere-forming efficiency (%) = scored sphere number/total plating cells. The area of each sphere was analyzed using ImageJ.

### Syngeneic mouse model

Female C57BL/6J mice were purchased from Jackson Laboratory. All studies were approved and supervised by the Institutional Animal Cancer and Use Committees at Georgetown University. 5 × 10^5^ cancer cells (PT 1988, PTU 63174, or PTU 63175) were intraperitoneally injected into 7 weeks old female C57BL/6J mice. After 8 weeks, the mice were dissected, and tumor formation and ascites accumulation in peritoneal cavity were examined.

### TCGA and Kaplan–Meier analysis

Oncoprints and copy number alteration graph were generated using cBioPortal for Cancer Genomics. Briefly, oncoprints generate graphical representations of genomic alterations, somatic mutations, and mRNA expression changes. The following studies were used for the TCGA analysis: serous ovarian cancer (PanCancer Atlas), esophageal adenocarcinoma (PanCancer Atlas), Uterine carcinosarcoma (PanCancer Atlas), head-and-neck tumors (PanCancer Atlas), lung squamous cell carcinoma (PanCancer Atlas), and lung adenocarcinoma (PanCancer Atlas). Data were downloaded as log2 (norm_count+1). Data of USP13 expression in human normal tissues were obtained from Genotype-Tissue Expression (GTEx) database. Kaplan–Meier curves were estimated with the KM plotter. The KM plotter was used to analyze the overall, progression-free, and post-progression survival of ovarian cancer patients.

### Statistical analysis

Data are presented as mean ± SD. Survival rates for animal studies were analyzed by log-rank test, using GraphPad Prism software. *P* values were determined by a two-tailed Student’s t-test, unless otherwise specified, with *P* < 0.05 considered statistically significant.

## Supplementary information

Supplemental Material
