# Supplementary material for: USP13 promotes development and metastasis of high-grade serous ovarian carcinoma in a novel mouse model
Source: Oncogene. 2022 Feb 16;41(13):1974–85. doi: 10.1038/s41388-022-02224-x (PMC8956511; doi:10.1038/s41388-022-02224-x)
Supplement: Supplementary file 1 — Supplemental Material [file 41388_2022_2224_MOESM1_ESM.pdf]

Supplementary Information

- Supplementary Figure Legends
- Supplementary Figure 1-4
- Supplementary Table 1.

Supplementary Figure 1. *USP13* expression in normal human tissues and *USP13*-amplified human cancers. (A) *USP13* mRNA expression level in normal human tissues (GTE database). **(B)** The percentage of genomic alterations of *USP13* in esophageal squamous carcinoma (ESCC), head and neck squamous cell carcinoma (HNSCC), endometrial carcinoma (EC), and non-small cell lung cancer (NSCLC). **(C)** Correlation between the copy number alteration and mRNA expression of USP13 in various cancers.

Supplymentary figure 1

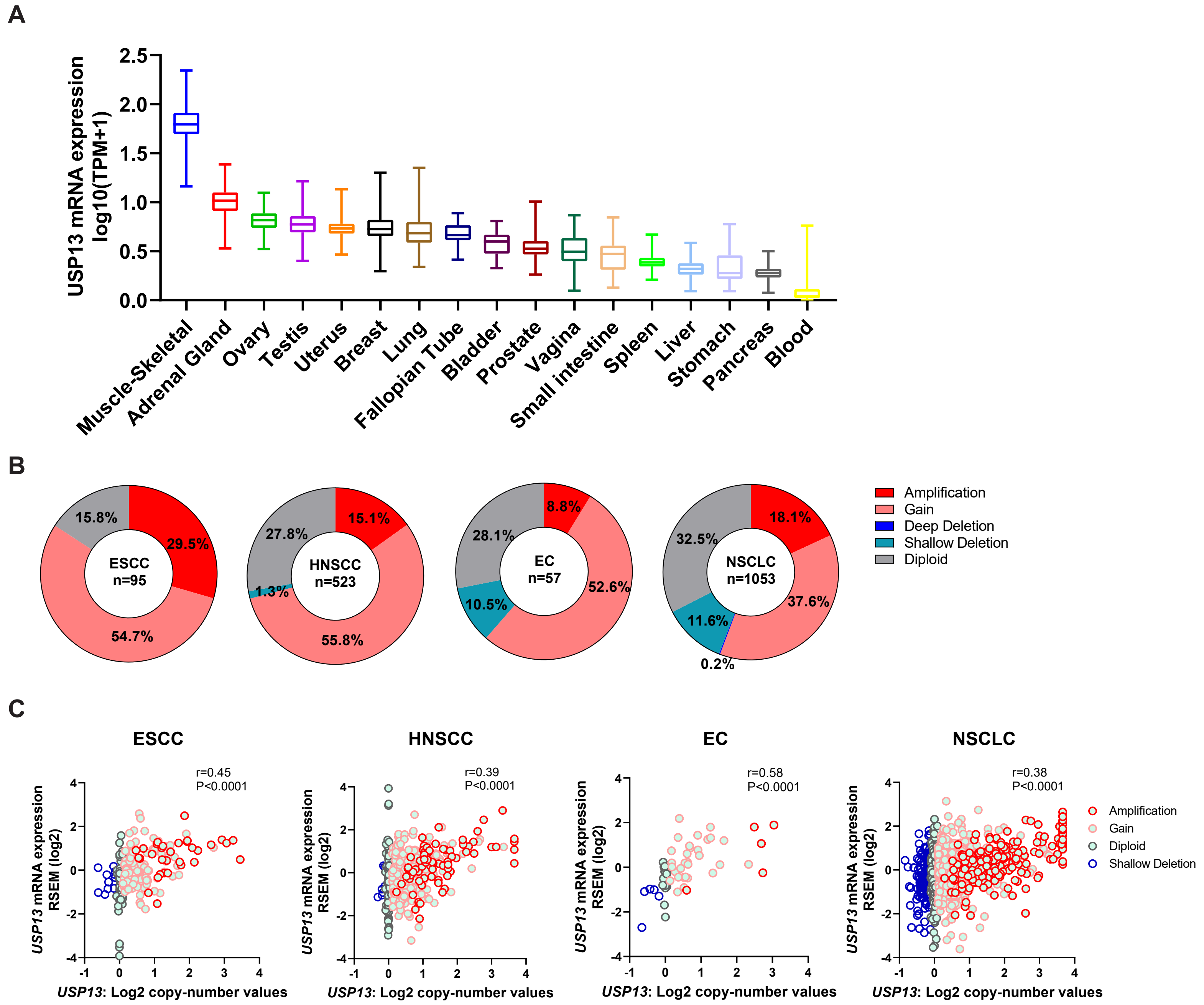

Supplementary Figure 2. Generation of conditional USP13 knock-in mouse model (A) Diagram of targeted knock-in (KI) of *Usp13*-LoxP-Stop-LoxP into Rosa26 locus, which allows conditional mouse *Usp13* mRNA expression controlled by Cre-mediated recombination. **(B)** Genotyping PCR (F1/R1) with genomic DNA extracted from the tail of heterozygous (KI/WT) and wild-type (WT/WT) mice. **(C)** Southern blotting assay confirmed knock-in allele (WT- 6.07 kb, KI-7.62 kb). **(D)** OSE-specific Cre-mediated recombination by AdCre ovarian intrabursal injection. PCR amplification of Cre recombinase gene and Cre-mediated recombined *Trp53* and *Pten* in a PT mouse after a week of AdCre injection. Genomic DNA was isolated from the 12 hours digestion of the following tissues: Liv-Liver, 1h Ov-Ovary (1 hour digestion), 12h Ov-Ovary (12 hours digestion), Ovt-Oviduct, and Ut-Uterus. **(E)** H&E staining and Pankeratin IHC in ovaries after 10 days with (+AdCre) or without AdCre (-AdCre) intrabursal injection from same PT mouse. White dot line indicates ovarian surface epithelial layer. Scale bars, 50 μm.

Supplymentary figure 2

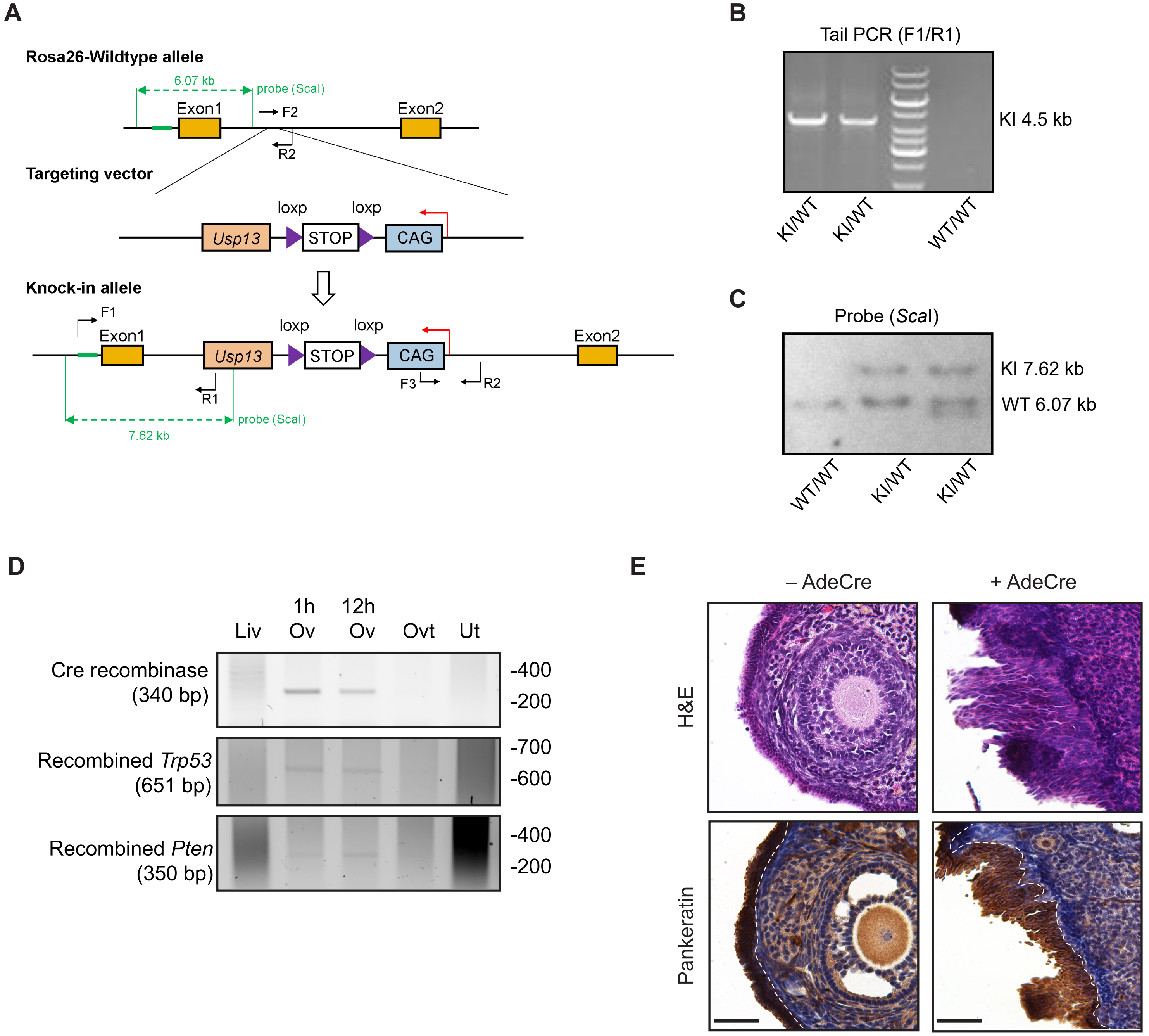

Supplementary Figure 3. Characterization of PT and PTU tumors. (A) Ovarian tumor development in PT and PTU mice at 69 days post AdCre viral injection. Left side is AdCre virus injected and right side is non-injected ovary. **(B)** USP13 induces hyperplasia in OSE of PU mouse. H&E stain and IHC analysis (Ki-67 and CK7) of ovaries in P and PU mice after 109 days of unilateral AdCre intrabursal injection. **(C)** Strong expression of USP13 was validated in primary ovarian tumors isolated from PTU mice (n=2) compared to PT mice (n=2). **(D)** High resolution of H&E stain image of Fig.4A. **(E)** IHC analysis of epithelial marker CK7 and the granulosa cell marker inhibin α. Both PT and PTU tumors were CK7-positive, but negative for Inhibin α. Scale bars, 50 μm.

Supplymentary figure 3

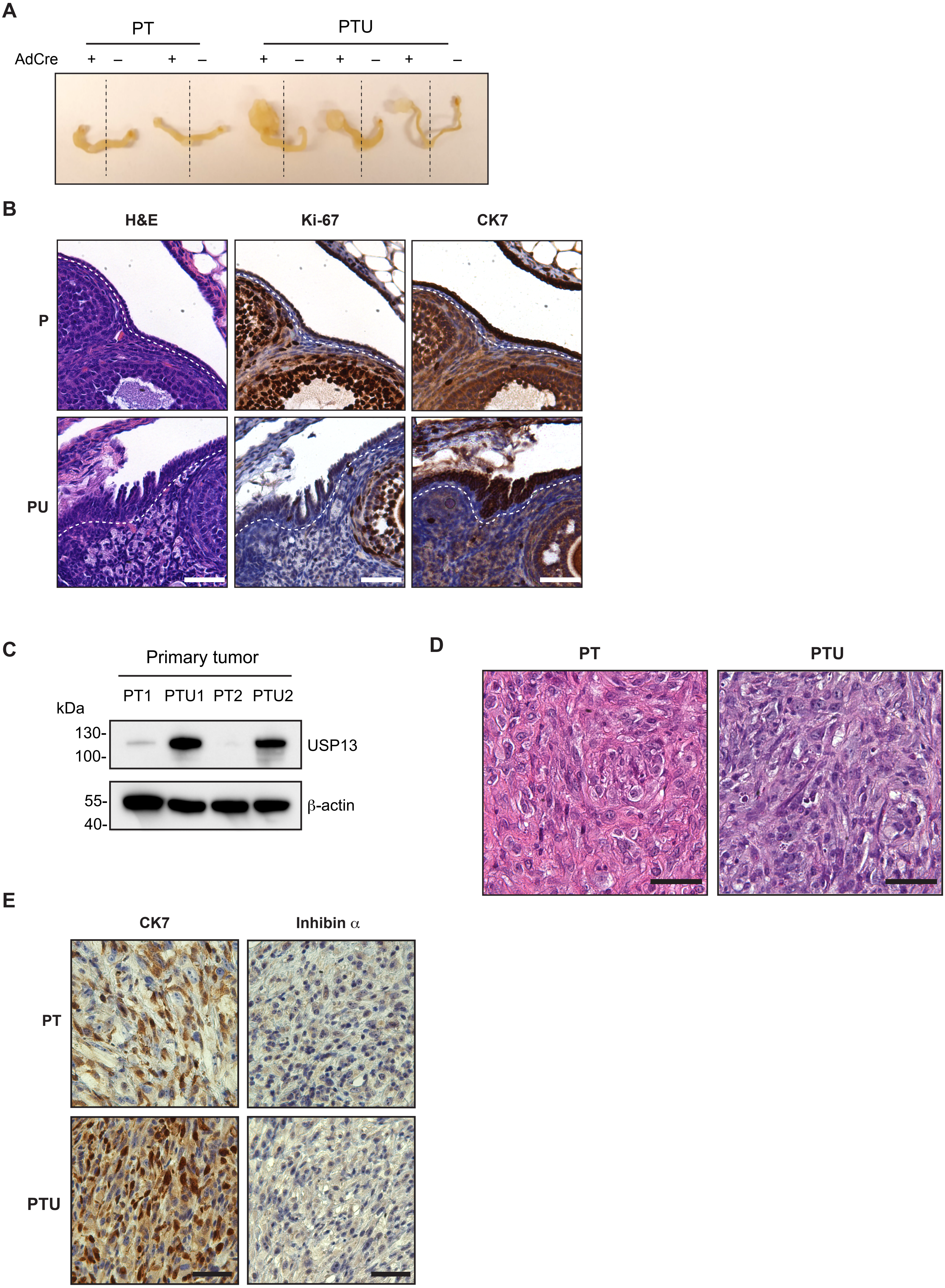

Supplementary Figure 4. Enhanced invasive and metastatic properties of PTU primary cancer cells in 3D culture and syngeneic mouse models. (A) Representative images of PT and PTU tumor spheroids showing invasive phenotypes in a solidified Matrigel culture dome. Arrows indicate protrusions from PTU spheroids. Scale bars = 50 μm. **(B)** PTU tumor spheroids with culture medium containing 2% Matrigel in an ultra-low attachment plate. PTU spheroids show invasive features (arrow) like protrusions and dissemination. Black scale bars = 250 μm, grey scale bars = 50 μm. **(C)** Abdominal cavity of wild-type C57BL/6 mouse after intraperitoneal (I.P) injection of $5x10^5$ PT or PTU cells, showing that PTU cells caused ascites formation and large tumor masses in omentum (i, iv), mesentery (ii, v), peritoneal wall (iv), and diaphragm (iii).

Supplymentary figure 4

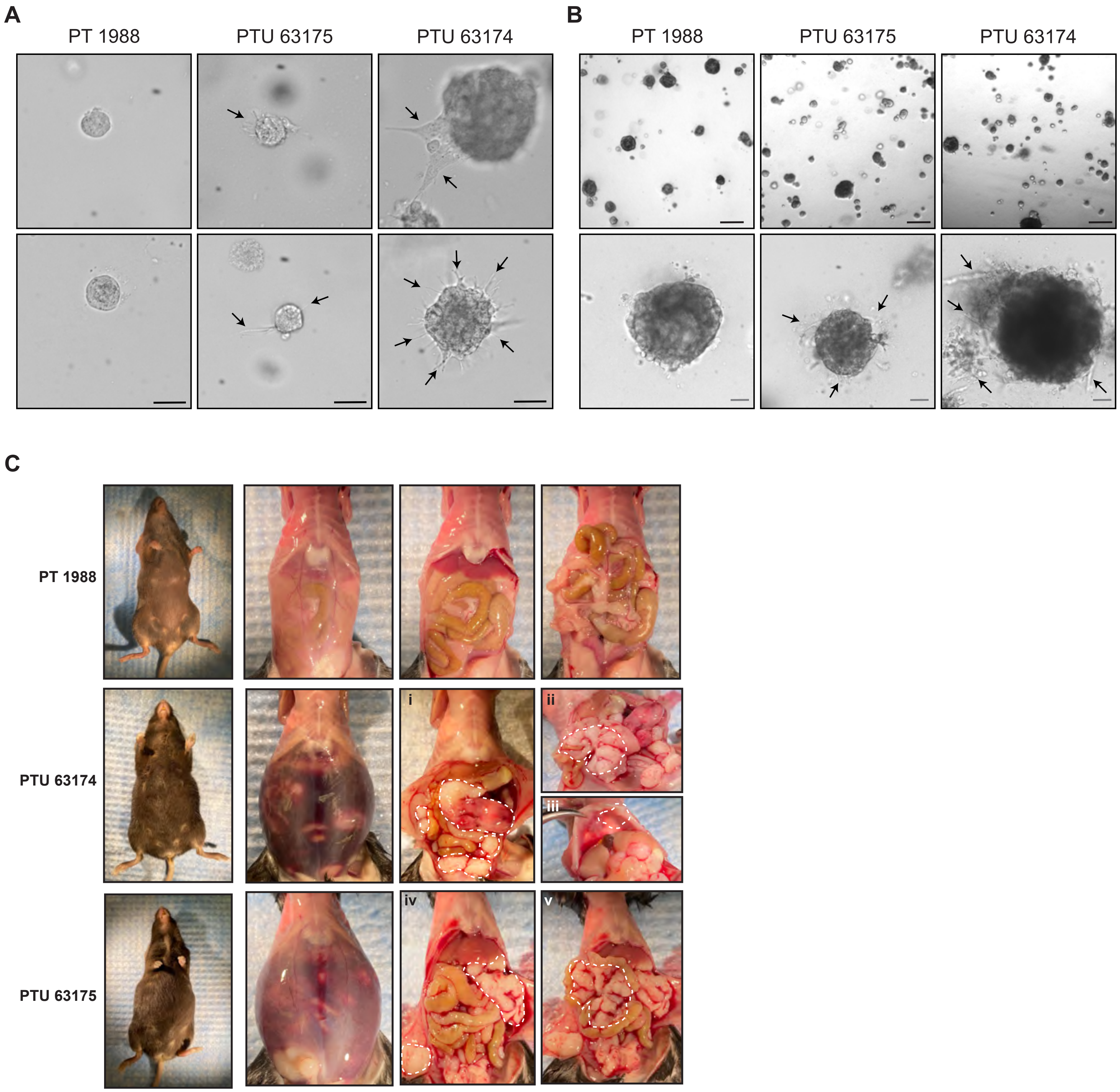

Supplementary Table 1. Primers used in this study

Name	Sequence (5'-3')	Purpose
CreFW	GAACCTGATGGACATGTTCAGG	*Cre* gDNA PCR
CreRV	AGTGCGTTCGAACGCTAGAGCCTG T	
PtenCreFW	ACTCAAGGCAGGGATGAGC	Recombined *Pten* gDNA PCR
PtenCreRV	GCTTGATATCGAATTCCTGCAGC	
Trp53CreFW	CACAAAAACAGGTTAACCCAG	Recombined *Trp53* gDNA PCR
Trp53CreRV	GAAGACAGAAAAGGGGAGGG	
USP13 F1	CACTTGCTCTCCCAAAGTCGCTC	*Usp13* genotyping
USP13 F2	AGATGTACTGCCAAGTAGGAAAGTC	
USP13 R	ATACTCCGAGGCGGATCACAA	
Pten Fw	CAAGCACTCTGCGAACTGAG	*Pten* genotyping
Pten Rv	AAGTTTTTGAAGGCAAGATGC	
Trp53 Fw	GGTTAAACCCAGCTTGACCA	*Trp53* genotyping
Trp53 Rv	GGAGGCAGAGACAGTTGGAG	
